# The Seroprevalence and Seropositivity of SARS-CoV-2 among Healthcare Workers during the Third Pandemic Wave

**DOI:** 10.3390/antib12010002

**Published:** 2022-12-23

**Authors:** Atefeh Vaezi, Hamed Fakhim, Saeed Abbasi, Soraya Masoudi, Mahnaz Hosseini Rizi, Shaghayegh Haghjooy Javanmard

**Affiliations:** 1 Cancer Prevention Research Center, Isfahan University of Medical Sciences, Isfahan 8174673461, Iran; 2 Infectious Diseases and Tropical Medicine Research Center, Isfahan University of Medical Sciences, Isfahan 8174673461, Iran; 3 Anesthesiology and Critical Care Research Center, Nosocomial Infection Research Center, Isfahan University of Medical Sciences, Isfahan 8174673461, Iran; 4 Department of Physiology, Applied Physiology Research Center, Cardiovascular Research Institute, Isfahan University of Medical Sciences, Isfahan 8174673461, Iran

**Keywords:** SARS-CoV-2, COVID-19, healthcare worker, seroconversion, seroepidemiology, seroprevalence

## Abstract

Background: Due to the unclear protective role of produced antibodies and the need for seroepidemiologic studies, we surveyed the COVID-19 seroprevalence among healthcare professionals who had direct or indirect contact with COVID-19 patients. Methods: From 19 October 2020 to 17 February 2021, 300 healthcare workers were enrolled and tested for serum antibodies in this prospective cohort study. Demographic information, risk factors, and infection history were collected. Anti- SARS-CoV-2 IgG and IgM antibody titers were determined to estimate the seroconversion rate. Results: During the first and second phases of the study, the positive seroconversion rates were 31.7 and 26.6%, respectively. In seronegative individuals, sixteen (10.6%) new cases of COVID-19 and five (6.3%) reinfections were identified. Among those with a positive antibody level, forty-one (36.9%) healthcare workers reported no symptoms in the preceding months. There was no association between occupational exposure and an increased probability of seroconversion. Conclusions: The seropositivity rate and the rate of asymptomatic individuals with seroconversion was remarkable and could be an indicator of a high infection rate among healthcare workers.

## 1. Introduction

SARS-CoV-2 had been responsible for approximately 643 million infections and 6.6 million deaths worldwide by December 2022 [1]. Currently, the real-time reverse transcriptase polymerase chain reaction (RT-qPCR) is the gold-standard test for detecting SARS-CoV-2. However, after 21 days, the test’s sensitivity drops from over 90% to 30% [2]. On the other hand, infection control strategies targeted symptomatic individuals, and asymptomatic individuals remain undiagnosed [3,4]. Consequently, antibody testing allows those asymptomatic, or symptomatic but with a negative PCR, to be identified [5].

Anti-SARS-CoV-2 immunoglobulin M (IgM) titer begins to increase during the first week of symptoms, reaches its peak 15–30 days after the onset of illness, and subsequently decreases. Immunoglobulin G (IgG) titer increases during the second week of infection, peaks 20 days after the onset of illness, and persists for at least two months [6]. Consequently, IgM serves as a marker for the acute phase of the disease, whereas IgG is essential for long-term immunity [7,8]. Detecting previous infection and immunity to COVID-19 is an important epidemiological issue, and serological tests can assist in this situation [9].

Healthcare workers (HCWs) demonstrate a higher risk of contracting the disease than the general population. Those with direct contact with COVID-19 patients have a 2.13 to 11.6-fold increase in the risk of infection compared to other HCWs and the general population [10,11,12,13,14]. Moreover, studies have uncovered a high prevalence of asymptomatic individuals ranging from 18% to 81% [15].

This study aims to evaluate the seroprevalence of IgG and IgM antibodies against SARS-CoV-2 in HCWs in a COVID-19-designated hospital in Isfahan, Iran, before and after the third wave of the pandemic.

## 2. Materials and Methods

This prospective cohort study was conducted between 19 October 2020, the start of the third wave, and 17 February 2021, the end date of the third wave, in Iran at Alzahra hospital, which comprises 950 beds and nearly 2500 HCWs affiliated with Isfahan University of Medical Sciences, Isfahan, Iran. 

### 2.1. Hospital Settings

Alzahra hospital was specifically designated for COVID-19 patients at the beginning of the third wave. All routine activities were discontinued, and all medical and surgical wards were converted into COVID-19 wards. These circumstances gave the researchers the opportunity to investigate the impact of the pandemic on HCWs before and after the third COVID-19 wave. This study was approved by the Ethics Committee of the Isfahan University of Medical Sciences (IR.MUI.MED.REC.1399.860). All participants were informed of the study objectives and provided written consent before participation. HCWs participated voluntarily and they were allowed to discontinue the study at any time. Data were managed anonymously.

### 2.2. Study Design and Participants

Two phases of data collection were conducted: the first, from 19 October to 28 October 2020, was concurrent with the start of the COVID-19 third wave, and the second, from 14 January to 17 February 2021, was simultaneous with the end of the third wave. 

**Figure 1:**
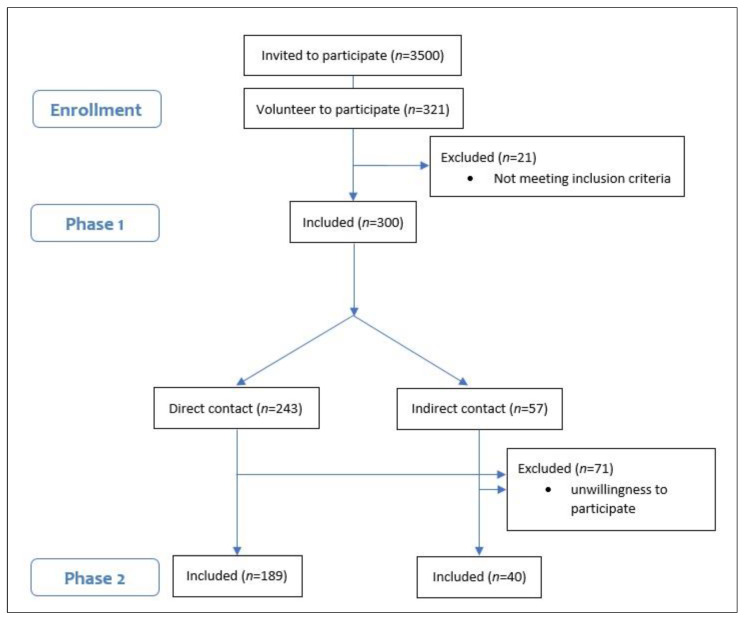
Flow diagram of participants.

Based on the Morgan table [16] for calculating sample size, 300 HCWs were included in the study by a convenience sampling method. An invitation describing the study’s objectives was sent to the hospital’s virtual groups and all hospital personnel with direct or indirect contact with COVID-19 patients were invited to participate. Administrative personnel were excluded from the study. HCWs were categorized according to their level of contact with COVID-19 patients (direct vs. indirect) and tracked through the third wave (almost 3–4 months). Seventy-one HCWs refused to participate in the second phase of the study (Figure 1).

Participants were asked to complete a questionnaire concerning demographic information, including age, gender (male and female), occupation (doctor, nurse, and others), level of care for COVID-19 patients (direct or indirect contact), educational level (high school and lower, bachelor’s degree, medical doctor and higher), and ward assignment (emergency, intensive care unit, and others). In addition, information regarding the presence of comorbidities (including height and weight, cardiovascular disease, cancer, respiratory disease, and diabetes) was collected. Furthermore, participants were asked if they had received training on infection and prevention control (IPC) measures and if they adhered to IPC guidelines. They were also asked if they had a history of COVID-19 in the preceding months. Individuals with a history of COVID-19 were asked to report their symptoms (including fever, sore throat, cough, rhinorrhea, dyspnea, chills, nausea or vomiting, diarrhea, loss of appetite, anosmia or ageusia, skin rash, conjunctivitis, body or joint pain, fatigue, and headache).

### 2.3. Serologic Assay and Measures

In each phase, 5 mL of venous blood was collected in an ethylenediaminetetraacetic acid (EDTA)-coated microcontainer and transferred immediately to the Core Facility Laboratory at Isfahan University of Medical Sciences, Isfahan, Iran, where it was centrifuged, and the sera were separated. Serum samples were analyzed with SARS-CoV-2 ELISA kits (Pishtaz Teb, Iran; catalog numbers PT-SARS-CoV-2.IgG-96 and PT-SARS-CoV-2.IgM-96) with indirect method to determine the presence of SARS-CoV-2-specific IgG and IgM antibodies; the kits were coated with Nucleocapsid (N) antigene [17,18]. The Iran Food and Drug Administration approved the kits. The manufacturer-reported sensitivity and specificity of ELISA kits are 94.1% and 98.3% for the SARS-CoV-2 IgG and 79.4% and 97.3% for the SARS-CoV-2 IgM, respectively. Cut-off value for IgG was calculated as the mean optical density (OD) value of the negative control plus 0.15. Cut-off value for IgM was calculated as the OD value of negative control plus 0.25. By dividing the OD of samples by the cut-off value, the cut-off index was calculated. According to the manufacturer’s instructions, a cut-off index of less than 0.9 is considered negative, 0.9–1.1 is considered suspect, and greater than 1.1 is considered positive. Seropositivity was considered a positive result in IgM or IgG, or both. As IgM increases, typically, in first days of infection and is referred as an indicator for acute or recent infection [19], we consider the elevation of IgM at follow-up in cases that had elevated levels of IgG but not IgM at baseline as reinfection.

### 2.4. Statistical Analysis

Data were analyzed using SPSS version 21.0.1 (SPSS Inc., Chicago, IL, USA). To test the normality, the Kolmogorov–Smirnov test was applied. Age was expressed as mean and standard deviation (SD). Qualitative variables were described using frequency tables. For presenting the anti-SARS-CoV-2 IgM and IgG, the geometric titer (GMT) was used. In order to compare demographic variables between the two groups, an independent *t*-test and chi-square were employed. The seroconversion rate between groups was compared using a chi-square test. To compare GMT between groups and before and after the study, a Mann–Whitney U test and a Wilcoxon test were applied, respectively. The *p*-value of <0.05 assumed as significant.

## 3. Results

This study included 300 HCWs employed at Alzahra hospital, including 211 (70.3%) nurses and 32 (10.7%) doctors/medical students. The normality hypothesis was checked for age using Kolmogorov–Smirnov, which indicated no evidence of violation of the assumption (*p*-value > 0.05). The study population’s mean age (±SD) was 38.5 ± 9.15 (median: 38).

HCWs were grouped based on their level of contact with COVID-19 patients: direct contact (*n* = 243, 81%) and indirect (*n* = 57, 19%). There were no statistically significant differences between the two groups regarding age, gender, or comorbidities. All physicians and nurses were in the direct contact group. Most HCWs with direct contact (56.8%) worked in the ICU, compared to 35.1% of HCWs with indirect contact (*p*-value = 0.01). Education level was also different between the two groups (*p*-value < 0.001). The level of IPC education did not differ between the two groups, but the level of IPC compliance was significantly higher in the indirect contact group than in the direct contact group (80.7 vs. 66.7; *p*-value = 0.03) (Table 1).

### 3.1. Serologic Findings and Seroconversion Rate

**Figure 2:**
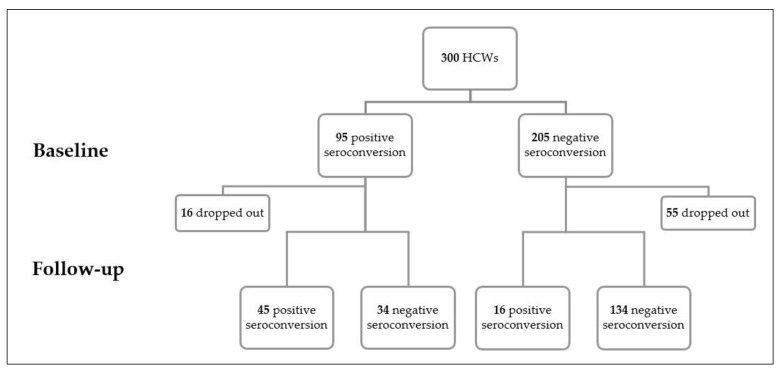
The positive and negative seroconversion at baseline and follow-up.

The overall seroconversion rate was 37.0% (111/300). At baseline, 31.7% of HCWs (95/300) exhibited positive levels of IgG or IgM or both. Seventy-one individuals dropped out and the seropositivity rate at follow-up was 26.6% (61/229). During follow-up, 43% (34/79) of HCWs with a positive level of antibodies became seronegative, and 16 new seropositive HCWs (incidence: 16/150 = 10.6%) were identified (Figure 2). Even though 13/16 HCWs were in the direct contact group, the rate of new seropositive cases did not differ significantly between the two groups (*p*-value = 0.5). Among those with positive IgG and negative IgM levels at baseline, five cases exhibited a positive IgM level at the follow-up time, indicating reinfection (5/79; 6.3%).

At baseline and follow-up, the seroconversion rate did not differ between the two groups of direct and indirect contact (*p*-value = 0.1 and 0.2, respectively) (Table 2). A Wilcoxon test were used to compare changes in IgM and IgG serum levels at baseline and follow-up time. The mean of GMT for anti-SARS-CoV-2 IgM decreases from 0.37 (95% CI = 0.34–0.41) to 0.23 (95% CI = 0.21–0.24) (*p*-value < 0.001). For anti-SARS-CoV-2 IgG the mean of GMT decreases from 0.77 (95% CI = 0.67–0.89) to 0.56 (95% CI = 0.48–0.65) (*p*-value < 0.001).

At baseline, those with direct contact had a higher GM mean of IgM compared to the indirect contact group, but this difference was not statistically significant (0.38, 95% CI: 0.35–0.42 vs. 0.37, 95% CI: 0.31–0.43; *p*-value = 0.83). Additionally, there was no significant difference between the two groups at the follow-up time regarding IgM levels (*p*-value = 0.95).

**Figure 3:**
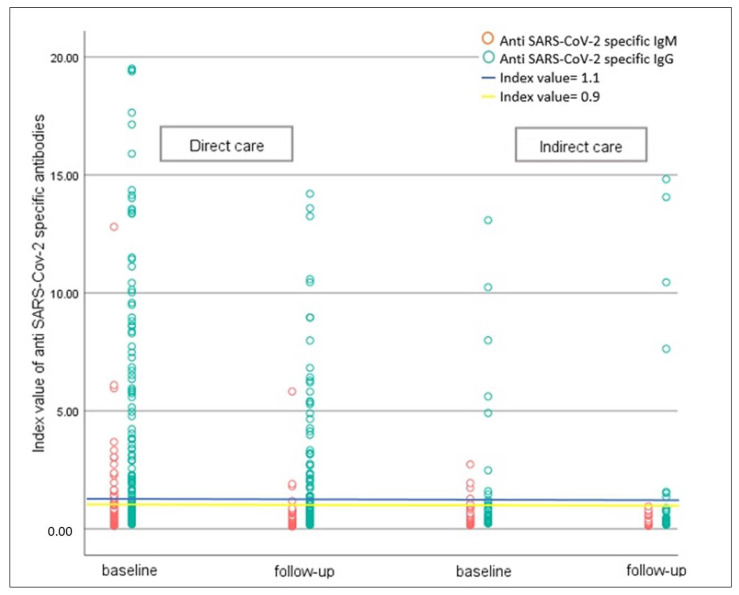
Index value of serum Anti SARS-CoV-2 specific IgG and IgM in two groups of direct and indirect contact; each circle represents one HCW. The blue and yellow lines represent cut-off index values of 1.1 and 0.9, respectively.

IgG, a marker of previous infection, was higher in the direct contact group (*p*-value = 0.02) at baseline, but this difference diminished over time. Figure 3 presents the index value for anti-SARS-CoV-2 specific IgG and IgM in direct and indirect contact groups at the baseline and the follow-up time.

### 3.2. Symptoms Based on Seroconversion

In the whole cohort, 122 (40.6%) HCWs reported one or more symptoms during previous months. From 111 HCWs who showed positive seroconversion, 41 (36.9%) did not report any symptoms in preceding months (Table 3).

The most prevalent symptoms in HCWs include fatigue (86.9%), body and joint pain (82.0%), and headaches (76.2%). In contrast, skin rash (12.3%) and conjunctivitis (10.7%) were the least prevalent symptoms. Considering seropositivity, three symptoms were more prevalent in the seropositive HCWs; 73.9% of HCWs in the seropositive group and 56.6% in the seronegative group report chills as one of their symptoms (*p*-value, 0.04); nausea or vomiting was reported by 48 (39.3%) HCWs and was higher in the seropositive HCWs than seronegative HCWs (49.3 vs. 26.4, respectively; *p*-value, 0.01); and anosmia or ageusia was more prevalent in the seropositive group in comparison to the seronegative group (62.3 vs. 32.1, respectively; *p*-value, 0.001) (Table 3).

## 4. Discussion

Antibody measurements are a crucial aspect of estimating the level of herd immunity in communities. Dynamic surveillance studies on the kinetics and stability of humoral immunity is useful in assessing the risk of reinfection and making decisions about the best time for antibody testing [5]. This prospective cohort study examined serum levels of anti-SARS-CoV-2 IgM and IgG in HCWs in Isfahan, Iran, according to their level of contact with COVID-19 patients during the third pandemic wave. By introducing and administering COVID-19 vaccines, studies on the stability of infection or vaccine-acquired antibodies aid policymakers in making sound decisions regarding the administration of booster doses of vaccines and setting priorities. Comparing the similarity of immunity induced by infection or COVID-19 vaccine, infection-induced immunity reveals more protection than non-recent vaccination, but less protection than a booster dose against hospitalization [20]. Additionally, previous infection with SARS-CoV-2 plays as a booster dose in fully vaccinated individuals [21].

Overall, 36.3% of HCWs have anti-SARS-CoV-2 antibodies. Through the third wave (almost 4 months follow-up), the incidence of new cases was 10.5% in our study population. The overall seroconversion rate among our HCWs was greater than that observed in previous studies. Houlihan et al. [22] reported a 20% seroconversion rate among frontline HCWs during the initial pandemic wave. Another study in Sweden indicated an overall seroconversion rate of 19% during the late phase of the first wave [23]. Antibody seropositivity in Iran’s general population was projected to reach 17.1% (95% CI: 14.6–19.5) by the end of April 2020 [24]. This difference may be attributable to several potential causes; first, our study was conducted during the third pandemic wave, whereas previous studies were conducted during the first wave. Second, it may be due to the degree of compliance with IPC regulations and the availability of personal protective equipment. 

The number of kits for SARS-CoV-2 IgG and IgM detection remains insufficient. Moreover, antibody detection kits for SARS-CoV-2 may exhibit cross-reactivity with other antibodies [25]. There is some evidence that COVID-19 patients’ IgG titers are rapidly declining. Two months after the onset of infection, 12.9% of symptomatic and 40% of asymptomatic individuals become seronegative for IgG, suggesting that the seroprevalence of SARS-CoV-2 is likely underestimated [26]. Even though 16 new cases were identified during this study’s follow-up period, the seroconversion rate decreased from 31.7% to 26.6%.

In our study, 43% of seropositive cases become seronegative after approximately 3–4 months. Although some studies raise questions about reinfection or reactivation of SARS-CoV-2 diagnosed by PCR, negative seroconversion should be a cause for concern, given the likelihood of reinfection [27,28]. In a study, SARS-CoV-2 reinfection was reported after approximately 80 days [29]. Another study demonstrates that antibodies are detectable for more than six months [30]. Ye et al. show a 9% reactivation of SARS-CoV-2 in patients discharged from hospital after two negative PCR tests [31]. The reinfection rate in our study was calculated based on the increase of IgM in cases with a positive IgG level at baseline, and was 6.3%. This assumption is not tested with real time PCR, but in situations with scarce resources it could be an estimation of infection in the community.

Another aspect of our findings is the proportion of asymptomatic cases. A failure to control the spread of infection and break the chain may be attributable to asymptomatic carriers [32]. The asymptomatic carriage rate in studies varies from 0% to 100% based on sample size, sampling method, and country of study [33]. This should be considered, particularly for HCWs who have daily contact with COVID-19 patients and could transmit the virus to their families. In our study, 37% of HCWs with positive serologic tests reported no symptoms in the preceding months. The level of reaction against SARS-CoV-2 infection is an additional important aspect of being asymptomatic. A study describing the clinical and immunological characteristics of 37 asymptomatic patients revealed that they had lower levels of anti-inflammatory cytokines than symptomatic patients [26]. Early identification of suggestive symptoms could be referred as criterion for probable infection, and protective measures could put in place in this situation more quickly. We found a difference in the prevalence of COVID-19 reported symptoms between seropositive and seronegative HCWs. Chills, nausea and vomiting, and anosmia or ageusia were more prevalent among seropositive HCWs. Previous studies reported that COVID-19 symptoms and severity are highly associated with seroactivity [34,35].

The results of the present study showed no difference in the seropositivity rate between HCWs with direct or indirect contact. This could be explained by considering the infection’s source. The majority of our study’s new cases had direct contact. This is consistent with the findings of Sims et al., which indicate a higher rate of seropositivity among nurses, nursing assistants, respiratory therapists, and phlebotomists [36].

This study had several limitations. First, the study’s single-center design and relatively small study population, hindered by the unwillingness of some participants to participate in the follow-up period, made it difficult to draw more certain conclusions regarding the seroactivity of SARS-CoV-2. Second, to be confident about seroconversion and seropositivity we need to use the Western blot technique, but due to the availability of the ELISA method, and also financial limitations, the ELISA method was applied. Third, the questionnaires were self-administered and anonymous to respect participants’ confidentiality, leading to possible errors. Bias might have occurred if personnel at higher or lower risk for infection were less or more likely to volunteer to participate.

## 5. Conclusions

The results of this study reveal a positive seroconversion rate of 37% in HCWs at a COVID-19 designated hospital through the third wave of pandemic. The rate of asymptomatic individuals who were seropositive was 37% and the rate of negative seroconversion was 43% in our study population. We found that the rate of seropositivity is not associated with the level of care. Additionally, chills, nausea and vomiting, and anosmia or ageusia were more prevalent in seropositive HCWs. Longitudinal sero-surveillance studies, especially in high risk populations such as HCWs that have daily exposure with COVID-19 patients, could estimate the level of immunity in these populations. 

## Figures and Tables

**Table 1:** Demographic and clinical characteristics of study population.

	Total	Contact with COVID-19 Patients		*p*-Value
		Direct (*n* = 243)	Indirect (*n* = 57)	
Age (mean ± SD)	38.5 ± 9.15	38.2 ± 9.03	40.0 ± 9.60	0.19 *
Gender				0.1
Female	202 (67.3)	168 (69.1)	34 (59.6)	
Male	98 (32.7)	75 (30.9)	23 (40.4)	
Occupation				<0.001
Doctor/Resident/Medical student	32 (10.7)	32 (13.2)	0	
Nurse	211 (70.3)	211 (68.8)	0	
Others	57 (19.0)	0	57 (100)	
Ward				0.01
Emergency room	29 (9.7)	21 (8.6)	8 (14.0)	
ICU	158 (52.7)	138 (56.8)	20 (35.1)	
Others	113 (37.7)	84 (34.6)	29 (50.9)	
Education level				<0.001
High school and less	68 (22.7)	43 (17.7)	25 (43.9)	
Bachelor	198 (66.0)	167 (68.7)	31 (54.4)	
MD and more	34 (11.3)	33 (13.6)	1 (1.8)	
Educating about IPC				0.1
Yes	260 (86.7)	214 (88.1)	46 (80.7)	
No	40 (13.3)	29 (11.9)	11 (19.3)	
IPC guidelines compliance				0.03
Yes	208 (69.3)	162 (66.7)	46 (80.7)	
No	92 (30.7)	81 (33.3)	11 (19.3)	
Comorbidities				
CVD	13 (4.3)	10 (4.1)	3 (5.3)	0.4
Cancer	9 (3.0)	8 (3.3)	1 (1.8)	0.4
Respiratory diseases	2 (0.7)	1 (0.4)	1 (1.8)	0.3
DM	12 (4.0)	9 (3.7)	3 (5.3)	0.7
Any previous diseases	64 (21.3)	51 (21.0)	13 (22.8)	0.7
BMI ≥ 25	130 (45.5)	104 (44.4)	26 (50.0)	0.4

Data are presented in N (%) unless otherwise specified. *p*-value < 0.05 was considered as significant. * *p*-value was calculated using independent *t*-test. Others were calculated by chi-square test. Abbreviations: COVID-19, coronavirus disease 2019; ICU, intensive care unit; MD, medical degree; IPC, infection and prevention control; CVD, cardiovascular disease; DM, diabetes mellitus; BMI, body mass index.

**Table 2:** Serologic findings of the study population.

		Whole Sample	Contact with COVID-19 Patients		*p*-Value
			Direct (*n* = 243)	Indirect (*n* = 57)	
Baseline	Seroconversion *n* (%)				0.1 *
	Yes	95 (31.7)	82 (33.7)	13 (22.8)	
	No	205 (68.3)	161 (66.3)	44 (77.2)	
	Anti SARS-CoV-2 IgM	0.37 (0.34–0.41)	0.38 (0.35–0.42)	0.37 (0.31–0.43)	0.83 **
	Anti SARS-CoV-2 IgG	0.77 (0.67–0.89)	0.89 (0.71–0.99)	0.54 (0.41–0.70)	0.02 **
Follow-up	Seroconversion *n* (%)				0.2 *
	Yes	61 (26.6)	53 (28.0)	8 (20.0)	
	No	168 (73.4)	136 (72.0)	32 (80.0)	
	Anti SARS-CoV-2 IgM	0.23 (0.21–0.24)	0.22 (0.21–0.24)	0.24 (0.20–0.28)	0.95 **
	Anti SARS-CoV-2 IgG	0.56 (0.48–0.65)	0.58 (0.49–0.68)	0.49 (0.34–0.72)	0.32 **

Data are presented in geometric mean (95% CI) unless otherwise specified. *p*-values were calculated using * chi-square or ** Mann–Whitney U test. *p*-value < 0.05 were considered as significant. Abbreviations: COVID-19, coronavirus disease 2019; IgM, Immunoglobulin M; IgG, Immunoglobulin G.

**Table 3:** Frequency of reported symptoms in preceding months in HCWs based on seropositivity.

	Total (*n* = 122)	Seroconversion		*p*-Value
		Positive (*n* = 69)	Negative (*n* = 53)	
Fever (≥38)	63 (51.6)	41 (59.4)	22 (41.5)	0.05
Sore throat	68 (55.7)	40 (58.0)	28 (52.8)	0.5
Cough	71 (58.2)	42 (60.9)	29 (54.7)	0.4
Rhinorrhea	55 (45.1)	34 (49.3)	21 (39.6)	0.2
Dyspnea	58 (47.5)	35 (50.7)	23 (43.4)	0.4
Chills	81 (66.4)	51 (73.9)	30 (56.6)	0.04
Nausea/vomiting	48 (39.3)	34 (49.3)	14 (26.4)	0.01
Diarrhea	40 (32.8)	24 (34.8)	16 (30.2)	0.5
Loss of appetite	65 (53.3)	41 (60.3)	24 (45.3)	0.1
Anosmia or ageusia	60 (49.2)	43 (62.3)	17 (32.1)	0.001
Skin rash	15 (12.3)	8 (11.6)	7 (13.2)	0.7
Conjunctivitis	13 (10.7)	6 (8.7)	7 (13.2)	0.5
Body/joint pain	100 (82.0)	57 (82.6)	43 (81.1)	0.8
Fatigue	106 (86.9)	61 (88.4)	45 (84.9)	0.5
Headache	93 (76.2)	52 (75.4)	41 (77.4)	0.7

Data are presented as *n* (%). Data were analyzed using chi-square. Significance level set at *p*-value < 0.05.

## Data Availability

Data reported in this study are available via contacting the corresponding author.

## References

[1] WHO Coronavirus (COVID-19) Dashboard. https://covid19.who.int/.

[2] Miller T.E., Beltran W.F.G., Bard A.Z., Gogakos T., Anahtar M.N., Astudillo M.G., Yang D., Thierauf J., Fisch A.S., Mahowald G.K. (2020). Clinical sensitivity and interpretation of PCR and serological COVID-19 diagnostics for patients presenting to the hospital. FASEB J..

[3] Arons M.M., Hatfield K.M., Reddy S.C., Kimball A., James A., Jacobs J.R., Taylor J., Spicer K., Bardossy A.C., Oakley L.P. (2020). Presymptomatic SARS-CoV-2 infections and transmission in a skilled nursing facility. N. Engl. J. Med..

[4] Oran A.D.P., Topol E.J. (2020). Prevalence of Asymptomatic SARS-CoV-2 Infection: A Narrative Review. Ann. Intern. Med..

[5] Maine G.N., Lao K.M., Krishnan S.M., Afolayan-Oloye O., Fatemi S., Kumar S., VanHorn L., Hurand A., Sykes E., Sun Q. (2020). Longitudinal characterization of the IgM and IgG humoral response in symptomatic COVID-19 patients using the Abbott Architect. J. Clin. Virol..

[6] Xiao T., Wang Y., Yuan J., Ye H., Wei L., Liao X., Wang H., Qian S., Wang Z., Liu L. (2021). Early viral clearance and antibody kinetics of COVID-19 among asymptomatic carriers. Front. Med..

[7] Racine R., Winslow G.M. (2009). IgM in microbial infections: Taken for granted?. Immunol. Lett..

[8] Toulis P. (2021). Estimation of COVID-19 prevalence from serology tests: A partial identification approach. J. Econom..

[9] Sotgiu G., Barassi A., Miozzo M., Saderi L., Piana A., Orfeo N., Colosio C., Felisati G., Davì M., Gerli A.G. (2020). SARS-CoV-2 specific serological pattern in healthcare workers of an Italian COVID-19 forefront hospital. BMC Pulm. Med..

[10] Ng K., Poon B.H., Puar T.H.K., Quah J.L.S., Loh W.J., Wong Y.J., Tan T.Y., Raghuram J. (2020). COVID-19 and the risk to health care workers: A case report. Ann. Intern. Med..

[11] Lai X., Wang M., Qin C., Tan L., Ran L., Chen D., Zhang H., Shang K., Xia C., Wang S. (2020). Coronavirus disease 2019 (COVID-2019) infection among health care workers and implications for prevention measures in a tertiary hospital in Wuhan, China. JAMA Netw. Open.

[12] Nguyen L.H., Drew D.A., Graham M.S., Joshi A.D., Guo C.-G., Ma W., Mehta R.S., Warner E.T., Sikavi D.R., Lo C.-H. (2020). Risk of COVID-19 among front-line health-care workers and the general community: A prospective cohort study. Lancet Public Health.

[13] Korth J., Wilde B., Dolff S., Anastasiou O.E., Krawczyk A., Jahn M., Cordes S., Ross B., Esser S., Lindemann M. (2020). SARS-CoV-2-specific antibody detection in healthcare workers in Germany with direct contact to COVID-19 patients. J. Clin. Virol..

[14] Mansour M., Leven E., Muellers K., Stone K., Mendu D.R., AJJogim W. (2020). Prevalence of SARS-CoV-2 antibodies among healthcare workers at a tertiary academic hospital in New York City. J. Gen. Intern. Med..

[15] Nikolai L.A., Meyer C.G., Kremsner P.G., Velavan T.P. (2020). Asymptomatic SARS Coronavirus 2 infection: Invisible yet invincible. Int. J. Infect. Dis..

[16] Krejcie R.V., Morgan D.W. (1970). Determining sample size for research activities. Educ. Psychol. Meas..

[17] Pishtaz Teb Diagnostics SARS-CoV-2 IgG ELISA kits. http://pishtazteb.com/en/sars-cov-2-igg-elisa-kit/.

[18] Pishtaz Teb Diagnostics SARS-CoV-2 IgM ELISA kits. https://pishtazteb.com/en/sars-cov-2-igm-elisa-kit/.

[19] Fonseca M.H., Silva M.F., Pinto A.C., de Melo A.C., de Oliveira F.D., Araújo F.M., de Andrade L.O. (2022). Persistently positive SARS-CoV-2-specific IgM during 1-year follow-up. J. Med. Virol..

[20] Waxman J.G., Makov-Assif M., Reis B.Y., Netzer D., Balicer R.D., Dagan N., Barda N. (2022). Comparing COVID-19-related hospitalization rates among individuals with infection-induced and vaccine-induced immunity in Israel. Nat. Commun..

[21] Carazo S., Skowronski D.M., Brisson M., Sauvageau C., Brousseau N., Gilca R., Ouakki M., Barkati S., Fafard J., Talbot D. (2022). Estimated Protection of Prior SARS-CoV-2 Infection Against Reinfection with the Omicron Variant Among Messenger RNA–Vaccinated and Nonvaccinated Individuals in Quebec, Canada. JAMA Netw Open..

[22] Houlihan C.F., Vora N., Byrne T., Lewer D., Kelly G., Heaney J., Gandhi S., Spyer M., Beale R., Cherepanov P. (2020). Pandemic peak SARS-CoV-2 infection and seroconversion rates in London frontline health-care workers. Lancet.

[23] Rashid-Abdi M., Krifors A., Sälléber A., Eriksson J., Månsson E. (2021). Low rate of COVID-19 seroconversion in health-care workers at a Department of Infectious Diseases in Sweden during the later phase of the first wave; a prospective longitudinal seroepidemiological study. Infect. Dis..

[24] Poustchi H., Darvishian M., Mohammadi Z., Shayanrad A., Delavari A., Bahadorimonfared A., Eslami S., Javanmard S.H., Shakiba E., Somi M.H. (2021). SARS-CoV-2 antibody seroprevalence in the general population and high-risk occupational groups across 18 cities in Iran: A population-based cross-sectional study. Lancet Infect. Dis..

[25] Kubina R., Dziedzic A. (2020). Molecular and serological tests for COVID-19. A comparative review of SARS-CoV-2 coronavirus laboratory and point-of-care diagnostics. Diagnostics.

[26] Long Q.-X., Tang X.-J., Shi Q.-L., Li Q., Deng H.-J., Yuan J., Hu J.-L., Xu W., Zhang Y., Lv F.-J. (2020). Clinical and immunological assessment of asymptomatic SARS-CoV-2 infections. Nat. Med..

[27] Roy S. (2020). COVID-19 reinfection: Myth or truth?. SN Compr. Clin. Med..

[28] Falahi S., Kenarkoohi A. (2020). COVID-19 reinfection: Prolonged shedding or true reinfection?. New Microbes New Infect..

[29] Kellam P., Barclay W. (2020). The dynamics of humoral immune responses following SARS-CoV-2 infection and the potential for reinfection. J. Gen. Virol..

[30] Al-Naemi M.H.B., Hassanen W.S., El Nahrawi S.F.M., Rashad R.A. (2021). Life span of COVID-19 antibodies following infection in a sample worker population in Qatar. J. Emerg. Med. Trauma Acute Care.

[31] Ye G., Pan Z., Pan Y., Deng Q., Chen L., Li J., Li Y., Wang X. (2020). Clinical characteristics of severe acute respiratory syndrome coronavirus 2 reactivation. J. Infect..

[32] Yu X., Yang R. (2020). COVID-19 transmission through asymptomatic carriers is a challenge to containment. Influenza Other Respir. Viruses.

[33] Fakhim H., Nasri E., Aboutalebian S., Gholipour S., Nikaeen M., Vaezi A., Mousavi S., Faramarzi S., Farhang A., Javanmard S.H. (2021). Asymptomatic carriers of coronavirus disease 2019 among healthcare workers in Isfahan, Iran. Future Virol..

[34] Steensels D., Oris E., Coninx L., Nuyens D., Delforge M.-L., Vermeersch P., Heylen L. (2020). Hospital-wide SARS-CoV-2 antibody screening in 3056 staff in a tertiary center in Belgium. JAMA.

[35] Rudberg A.-S., Havervall S., Månberg A., Falk A.J., Aguilera K., Ng H., Gabrielsson L., Salomonsson A.-C., Hanke L., Murrell B. (2020). SARS-CoV-2 exposure, symptoms and seroprevalence in healthcare workers in Sweden. Nat. Commun..

[36] Sims M.D., Maine G.N., Childers K.L., Podolsky R.H., Voss D.R., Berkiw-Scenna N., Oh J., E Heinrich K., Keil H., Kennedy R.H. (2021). Coronavirus Disease 2019 (COVID-19) seropositivity and asymptomatic Rates in healthcare workers are associated with job function and masking. Clin. Infect. Dis..

